# Changes in the provision and utilisation of health care services for chronic health conditions during the COVID-19 pandemic in rural northeast South Africa: an interrupted time series analysis

**DOI:** 10.7189/jogh.15.04022

**Published:** 2025-01-31

**Authors:** Chodziwadziwa W Kabudula, Morelearnings Sibanda, Jessica Price, Jacques Du Toit, Nkosinathi Masilela, Kathleen Kahn, Francesc Xavier Gómez-Olivé, Susan Goldstein, Evelyn Thsehla, Micheal Kofi Boachie, Karen Hofman, Stephen Tollman

**Affiliations:** 1 SAMRC/Wits Rural Public Health and Health Transitions Research Unit (Agincourt), School of Public Health, Faculty of Health Sciences, University of the Witwatersrand, Johannesburg, South Africa; 2 Department of Epidemiology and Global Health, Umeå University, Umeå, Sweden; 3 SAMRC/WITS Centre for Health Economics and Decision Science – PRICELESS SA, School of Public Health, Faculty of Health Sciences, University of the Witwatersrand, Johannesburg, South Africa; 4 Discipline of Public Health Medicine, School of Nursing and Public Health, Howard College, University of KwaZulu-Natal, Durban, South Africa

## Abstract

**Background:**

The COVID-19 pandemic has impacted the provision and utilisation of health care services with varying magnitude across settings due to spatial temporal variation in the burden of COVID-19 cases and the roll-out of local COVID-19 response policies. This study assesses changes in the provision and utilisation of health care services for three major chronic health conditions (HIV/AIDS, hypertension, and diabetes) over the pre-COVID-19 and COVID-19 pandemic periods in a rural South African sub-district of Agincourt.

**Methods:**

Segmented interrupted time series regression models are applied to assess changes in the number of medication collection visits and new diagnoses for HIV/AIDS, hypertension, and diabetes from 1 January 2018 to 30 September 2021 covering the pre- COVID-19 period and the first three waves of the COVID-19 pandemic.

**Results:**

The number of medication collection visits for HIV/AIDS, hypertension, and diabetes dropped following the imposition of level 5 lockdown. Despite some improvements over the course of the pandemic, by the end of the third wave in September 2021, visits remained below the pre-COVID-19 era. The number of clinic visits for new diagnoses of HIV/AIDS and hypertension also fell after the introduction of level 5 lockdown. Although the number of new visits for HIV/AIDS bounced back to the pre-COVID-19 trends by the end of the third wave, the number of visits for new hypertension diagnoses remained significantly lower than expected. Referrals for collection of medications from the Central Chronic Medicines Dispensing and Distribution (CCMDD) programme, as an alternative to collection from clinics, increased exponentially over the course of the pandemic.

**Conclusions:**

Although the increased adoption of the CCMDD programme can in part account for decreased medication collection visits which persisted well after lockdown measures were lifted, marked reductions in the number of newly diagnosed cases of hypertension warrant concern. A deeper assessment of the appropriateness of referrals to the CCMDD programme as well as the longer-term effects on morbidity and mortality of missed treatment and/or delayed diagnosis is needed for a more granular understanding of the true ramifications of the COVID-19 pandemic and associated lockdown policies in the Agincourt subdistrict and other rural African settings.

The COVID-19 was declared a global pandemic by the World Health Organisation (WHO) on 11 March 2020 [1]. In response, governments in many countries implemented a set of compulsory measures popularly known as ‘lockdown’ that were applied wholesale to the general population in order to reduce COVID-19 transmission and protect high risk individuals [2]. In parallel, there was also an emergence and acceleration of new models of health care provision in many settings. Alternatives to in-person care such as telemedicine, decentralised renewal of scripts for chronic health conditions and non-clinic medication collection points were rapidly upscaled [3]. This was to balance the need to reduce the patient load at health care facilities in anticipation of an influx of acutely ill COVID-19 patients, whilst still providing continuity of care for patients with chronic health conditions and reducing their risk of contracting COVID-19.

In South Africa, there were five levels of lockdown measures implemented, with ‘level 5′ having the highest, most restrictive measures and ‘level 1’ having limited restrictive measures. Level 5 lockdown measures prohibited international and interprovincial travel and non-essential movement of people and ordered the shutting down of non-essential businesses, schools, and services[4–6]. During level 5 lockdown, individuals were ordered to remain in their place of residence except when ‘performing an essential service, obtaining an essential good or service, collecting a social grant, or seeking emergency, life-saving or chronic condition medical attention’. Level 1 lockdown measures permitted most normal activities to take place with precautions such as mask wearing with limitation of crowd size and adhering to health guidelines. Level 5 lockdown measures in South Africa were implemented on 27 March 2020 when the number of laboratory-confirmed case of COVID-19 had rapidly increased to 402 from one case on 5 March 2020. The lockdown levels were sequentially adjusted down to level 4 which permitted the opening of public transport on 1 May 2020, to level 3 which allowed some economic activity to resume on 1 June 2020, and finally to level 1 after the end of the first wave of the COVID-19 pandemic on 21 September 2020. The adjustment of levels of lockdown measures continued in 2021 in response to recorded infection rates during the second and third waves of the COVID-19 pandemic (**Figure 1**) [4].

**Figure 1:**
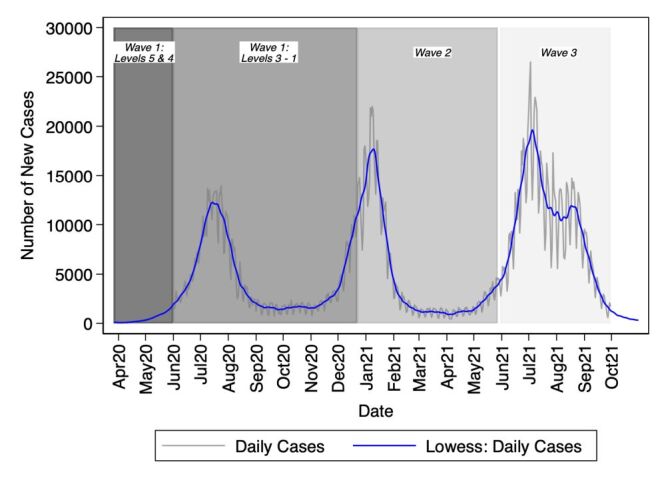
Evolution of COVID-19 pandemic in South Africa, March 2020 – September 2021.

Research studies have increasingly highlighted unanticipated consequences of COVID-19 related lockdown measures on customary utilisation of health care services for non-COVID-19 health conditions. Examples include reduced access to health care, an increase in missed visits for scheduled check-ups, a drop in childhood immunisation rates and reduced screening as well as delayed presentation and diagnosis of chronic health conditions such as hypertension, diabetes, HIV/AIDS and Tuberculosis [7–10]. However, their magnitudes have varied over time and across different settings due to the spatial temporal variations in the burden of COVID-19 cases and the roll-out of local COVID-19 response policies.

In South Africa, most studies assessing changes in the utilisation of health care services during the COVID-19 pandemic era only covered the first COVID-19 wave period [6,11–14]. There has also been little focus on assessing changes that occurred to the provision of health care services during the COVID-19 pandemic era. In addition, there is a paucity of studies using empirical data from rural settings. This study assesses changes to the provision and utilisation of health care services for three major chronic health conditions (HIV/AIDS, hypertension, and diabetes) over the first three waves of the COVID-19 pandemic (March 2020–September 2021). The study provides a longer view of the compounding transformation in the utilisation of health care services for chronic health conditions driven by the COVID-19 pandemic in one of South Africa’s rural settings. In addition, our study concurrently assesses changes in the number of patients with chronic health conditions enrolled in the Central Chronic Medicines Dispensing and Distribution (CCMDD) programme during the COVID-19 pandemic as an indicator of changes in the provision of health care services for chronic diseases in rural settings of South Africa. We focus on HIV/AIDS, hypertension and diabetes as they are the three most prevalent chronic health conditions in South Africa [15,16].

## METHODS

### Study design and setting

The population for this interrupted time series analysis study come from the Agincourt subdistrict located in a predominantly rural area of Bushbuckridge municipality in Mpumalanga Province, northeast South Africa. The area is underpinned by the long-running Agincourt Health and socio-Demographic Surveillance System (HDSS), which since 1992 provides a robust population denominator, with registration of all household members and rigorous collection of all vital events (deaths, births, movements) [17]. This former Bantustan area currently covering 31 villages is characterised by limited formal employment opportunities, widespread labour migration of men and increasingly women, and rapidly rising mortality from non-communicable conditions alongside the persistent burden from HIV/AIDS [18].

### Data sources

The study draws on two intersecting longitudinal data sources: the population-based HDSS with near-complete enrolment of all members of the Agincourt sub-district by age, sex/gender and household [17] and the Agincourt HDSS-clinic-hospital linkage system. The latter digitises longitudinal paper-based records from a network of eight public health care facilities making up the Agincourt subdistrict clinic platform to generate data on all chronic health care attendances, patient symptoms, examination findings, laboratory tests and medicines prescribed with linkage to the Agincourt HDSS data. This system also captures referrals to the CCMDD programme and medication collection schedules.

The roll-out of the CCMDD programme in the public health care system in South Africa started in 2016 in order to improve ease of access to medications for people living with chronic health conditions by reducing waiting times, congestion, and workload at health care facilities. The programme enrols patients whose chronic health condition is stable to have their medication delivered for collection at a point of their choosing monthly for a maximum of six months, after which a clinical review is mandated [19]. By October 2019 the CCMDD programme was in place in 88% of South Africa’s districts, with at least two million patients actively collecting their medication from the programme. About 64% of the patients utilising the programme were collecting only HIV/AIDS medications, 24% were collecting only non-HIV/AIDS related medication, and 12% were collecting both HIV/AIDS and non-HIV/AIDS related medications. Most notably, only 35% were collecting from non-health care/external pick-up points [20].

### Study outcomes

The study uses data on visits for collection of medications for HIV/AIDS, hypertension and diabetes from the eight primary health care facilities captured in the Agincourt HDSS-Clinic-Hospital Link System for individuals aged 18 years and visit schedules for collection of medications from the CCMDD programme for the same individuals. Outcomes evaluated include changes in the number of:

i) medication collection visits for HIV/AIDS, hypertension, and diabetes;

ii) new diagnoses of HIV/AIDS, hypertension and diabetes;

iii) visit schedules for collection of medications from the CCMDD programme over the period 1 January 2018 to 30 September 2021.

### Statistical analysis

Segmented interrupted time series regression models are used to assess changes in the number of medication collection visits and new diagnoses for HIV/AIDS, hypertension and diabetes. We divided time into five periods:

i) pre-lockdown from 1 January 2018 to the initial level 5 lockdown on 27 March 2020;

ii) initial level 5 and 4 lockdown period from 28 March through 31 May (*X_1_*);

iii) level 3–1 lockdown from 1 June through 22 December 2020 (*X_2_*);

iv) beginning until end of the second pandemic wave from 23 December 2020 through 27 May 2021 (*X_3_*);

v) beginning until end of the third wave from 28 May 2021 through 30 September 2021 (*X_4_*).

Analyses are stratified by sex (male and female) and age category (18–34,35–49,50–64, and 65 years and older). The age groups were chosen based on the age distribution of the patients captured in the Agincourt HDSS-Clinic-Hospital Link System that ensures sufficient numbers for the analysis.

In segmented regression models, the study period is partitioned into segments corresponding to the interventions (lockdowns) and it is assumed that there is a linear change over time in the specified outcome (number of medication collection visits or new diagnoses) within each time segment [21]. Segmented regression models used in our analyses take the form log(*Y_t_*) = *β_0_ + β_1_T + β_2_X_1_* + *β_3_X_1_ T_1_* + *β_4_X_2_* + *β_5_X_2_ T_2_* + *β_6_X_3_* + *β_7_X_3_ T_3_* + *β_8_X_4_* + *β_9_X_4_ T_4_* where *Y_t_* is the mean number of medication collection visits or new diagnoses at time point t (week for hypertension and HIV/AIDS and month for diabetes due to low numbers), *β_0_* is the number of medication collection visits or new diagnoses at baseline, *β_1_* is the pre-lockdown trend in the number of medication collection visits or new diagnoses and *β_2_* is the immediate change in the number of medication collection visits or new diagnoses at the time of the initial level 5 lockdown on 27 March 2020. The lockdown segments defined above are given by the dummy variables *X_1_*, …, *X_4_* coded 0 for the segment before the reference lockdown period and 1 for the period post the reference lockdown period. *T* is a linear term indicating the number of weeks (for hypertension and HIV/AIDS) or months (for diabetes to ensure sufficient numbers for the analysis) since the start of the study period and models the linear pre-lockdown trend while *T_1_*, …, *T_4_* represent the corresponding number of weeks (for hypertension and HIV/AIDS) or months (for diabetes) since the start of the lockdown segments *X_1_*, …, *X_4_* respectively. The regression coefficients *β_3_*, *β_5_*, *β_7_*, *β_9_* represent the level of change in the trends in the number of medication collection visits or new diagnoses during the lockdown segments *X_1_*, …, *X_4_* such that (*β_1_ + β_3_*), (*β_1_ + β_3_ + β_5_*), (*β_1_ + β_3_ + β_5_ + β_7_*), (*β_1_ + β_3_ + β_5_ + β_7_ + β_9_*) are trends during the lockdown segments *X_1_*, …, *X_4_* respectively. The regression coefficients from the Poisson model are exponentiated to obtain incidence rate ratios (IRR).

### Software

All analyses have been conducted using Stata version 17 (Stata Corp., College Station, USA).

### Ethics and consent

Ethical clearance for collection of individual and patient data into both the Agincourt HDSS, and HDSS-Clinic-Hospital Link system was obtained from the Human Research Ethics Committee (Medical) of the University of the Witwatersrand (protocol M151162). In particular, written informed consent was sought from all individuals to link their personal identifiers and clinical records from all involved clinics and hospitals with records held in the Agincourt HDSS population database.

### Patients and public involvement

The MRC/Wits Rural Public Health and Health Transitions Research Unit (Agincourt) which has been conducting population-based studies in the Agincourt sub-district since 1992 regularly engages with the public through its Public Engagement Office (PEO). The PEO ensures that the public understand research objectives and results and are able to raise concerns about the Unit’s research in their communities and provide feedback of research results at community meetings. The PEO works with members of the Community Advisory Group who ensures that information flows between the Agincourt Research Unit and the community, voices concerns, assesses the potential impact of the Unit’s research on the community, and maintains ongoing dialogue and consultation.

## RESULTS

A total number of 402 035 medication collection and 8779 new diagnosis visits for HIV/AIDS, hypertension and diabetes were captured from 21 066 patients into the Agincourt HDSS-Clinic-Hospital Link system in the eight primary health care facilities within the Agincourt subdistrict and HDSS during the period 1 January 2018 to 30 September 2021. Most patients (n = 15 649; 74.3%) were female. The distribution of patients by age was 26.3% aged 18–34 years, 35.0% aged 35–49 years, 22.5% aged 50–64 years and 16.1% aged 65 years or older. Most of the visits (244 275 medication collection and 4999 new diagnoses visits) were for HIV/AIDS followed by hypertension (123 513 medication collection and 1731 new diagnoses visits) and diabetes (21 379 medication collection and 228 new diagnoses visits) (**Table 1**). The largest absolute share of medication collection visits for HIV/AIDS was recorded from patients aged 35–49 years. However, most medication collection visits for hypertension and diabetes were recorded in patients 50 years or older.

**Table 1:** Average number of weekly medication collection and new diagnoses visits for HIV/AIDS, hypertension and diabetes in primary health care facilities, Agincourt subdistrict and HDSS, January 2018 – September 2021

Variables		HIV/AIDS				Hypertension				Diabetes		
		**New diagnoses**		**Medication collection**		**New diagnoses**		**Medication collection**		**New diagnoses**	**Medication collection**	
Number of visits		5522		253 119		2806		127 083		451	21 833	
Sex, n (%)												
*Female*		3997 (72.4)		191 181 (75.5)		1939 (69.1)		97 132 (76.4)		294 (65.2)	16 376 (75.0)	
*Male*		1525(27.6)		61 938 (24.5)		867 (30.9)		29 951 (23.6)		157 (34.8)	5457 (25.0)	
Age groups, n (%)												
*18–34 years*		2797 (50.7)		72 557 (29.7)		265 (9.4)		4760 (3.9)		26 (5.8)	651 (3.1)	
*35–49 years*		1842 (33.4)		106 959 (43.8)		859 (30.6)		26 026 (21.1)		84 (18.6)	3318 (15.5)	
*50–64 years*		726 (13.1)		49 841 (20.4)		979 (34.9)		43 973 (35.6)		203 (45.0)	8385 (39.2)	
*65+ years*		157 (2.8)		14 912 (6.1)		702 (25.0)		48 745 (39.5)		138 (30.6)	9021 (42.2)	
MD (IQR)*												
*Pre-covid*		30 (24–35)		1282 (1131–1397)		15 (13–19)		655 (607–696)		2 (2–3)	115 (107–121)	
*First Wave (L5-L4)*		13 (10–27)		1197 (1110–1307)		10 (5–11)		560 (519–621)		2 (1–3)	88 (81–110)	
*First Wave (L3-L1)*		28 (23–32)		1339 (1293–1448)		14 (13–17)		666 (630–712)		2 (2–3)	112 (96–118)	
*Second Wave*		28 (25–32)		1405 (1317–1466)		11 (9–14)		647 (622–673)		2 (1–4)	103 (98–111)	
*Third Wave*		22 (19–25)		1415 (1303–1430)		11 (8–15)		679 (654–699)		0 (0–0)	112 (100–121)	

IQR – Interquartile range (25th percentile–75th percentile), MD – median

*Median number of visits per week by lockdown period.

**Figure 2:**
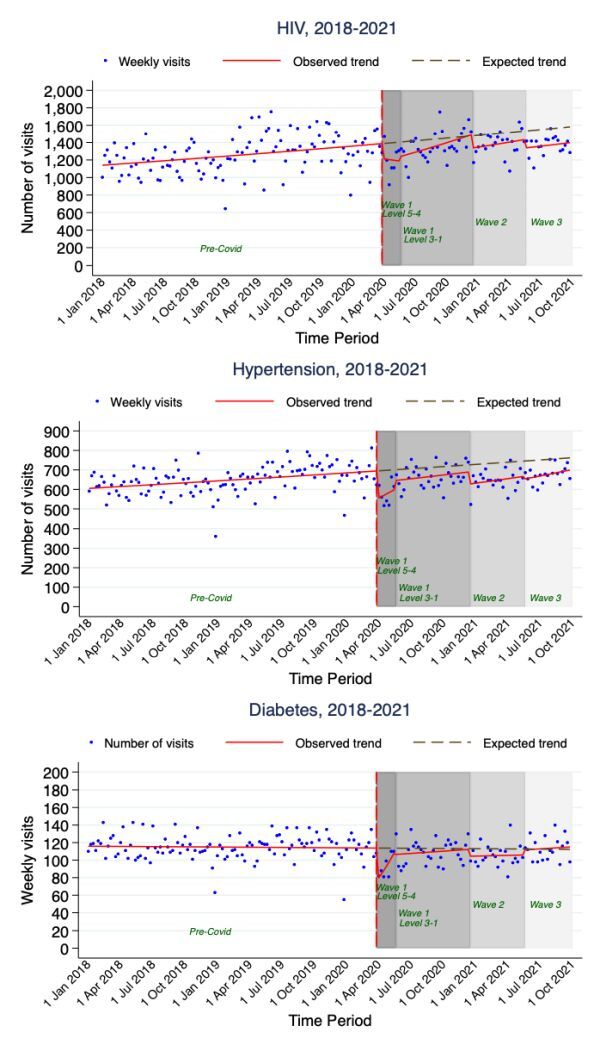
Trends in number of medication collection visits for HIV/AIDS, hypertension and diabetes.

**Figure 1** shows the evolution of the COVID-19 pandemic in South Africa from March 2020 to September 2021 and **Figure 2** and **Table 2** show trends in the number of medication collection visits for HIV/AIDS, hypertension and diabetes at different time periods of the COVID-19 pandemic from 1 January 2018 to 30 September 2021, overall and by age and sex. During the pre- COVID-19 period, the number of medication collection visits were increasing at an average rate of 0.17%, IRR = 1.0017 (95% confidence interval (CI) = 1.0015–1.0018) per week for HIV/AIDS and 0.12%, IRR = 1.0012 (95% CI = 1.001–1.0014) per week for hypertension but remained constant for diabetes. The weekly number of medication collection visits for these chronic conditions dropped significantly immediately after the introduction of the first level 5 lockdown visits dropped by 12.37%, IRR = 0.8764 (95% CI = 0.8347–0.9201) for HIV/AIDS, 20.85%, IRR = 0.7915 (95% CI = 0.7372–0.8497) for hypertension and 33.20%, IRR = 0.6680 (95% CI = 0.5568–0.8015) for diabetes.

**Table 2:** Trends in weekly number of chronic disease medication collection visits by condition, sex and age

Variables	Pre-lockdown trend, IRR (95% CI)	Immediate change at the start of lockdown, IRR (95% CI)	Level 5–4 trend, IRR (95% CI)	Level 3–1 trend, IRR (95% CI)	Second wave lockdown trend, IRR (95% CI)	Third wave lockdown trend, IRR (95% CI)
	exp*(β_1_)*	exp*(β_2_)*	exp*(β_1_* + *β_3_)*	exp*(β_1_* + *β_3_* + *β_5_)*	exp*(β_1_* + *β_3_* + *β_5_* + *β_7_)*	exp*(β_1_* + *β_3_* + *β_5_* + *β_7_* + *β_9_)*
HIV/AIDS	1.0017 (1.0015–1.0018)*	0.8764 (0.8347–0.9201)*	0.9969 (0.9863–1.0076)	1.0062 (1.0051–1.0074)*	1.0032 (1.0015–1.0050)*	1.0024 (1.0002–1.0046)*
Hypertension	1.0012 (1.0010–1.0014)*	0.7915 (0.7372–0.8497)*	1.0120 (0.9965–1.0277)	1.0022 (1.0006–1.0038)*	1.0028 (1.0002–1.0054)*	1.0037 (1.0006–1.0069)*
Diabetes	0.9998 (0.9993–1.0003)	0.6680 (0.5568–0.8015)*	1.0505 (1.0108–1.0917)*	1.0018 (0.9978–1.0058)	1.0006 (0.9942–1.0071)	1.0016 (0.9939–1.0093)
Females						
*18–34 years*	1.0011 (1.0008–1.0014)*	0.7628 (0.6925–0.8404)*	1.0200 (0.9989–1.0415)	1.0036 (1.0015–1.0058)*	1.0032 (0.9997–1.0067)	1.0018 (0.9974–1.0063)
*35–49 years*	1.0019 (1.0016–1.0021)*	0.8609 (0.7976–0.9292)*	0.9989 (0.9823–1.0158)	1.0066 (1.0048–1.0083)*	1.0026 (0.9999–1.0054)	1.0020 (0.9986–1.0055)
*50–64 years*	1.0018 (1.0015–1.0020)*	0.8726 (0.7991–0.9529)*	0.9947 (0.9756–1.0141)	1.0059 (1.0038–1.0079)*	1.0041 (1.0008–1.0074)*	1.0062 (1.0022–1.0102)*
*65+ years*	1.0006 (1.0002–1.0009)*	0.8843 (0.7941–0.9848)*	0.9918 (0.9686–1.0156)	1.0033 (1.0008–1.0058)*	1.0040 (1.0000–1.0080)	1.0038 (0.9990–1.0087)
Males						
*18–34 y*	1.0003 (0.9996–1.0010)	0.7693 (0.6036–0.9805)*	1.0265 (0.9743–1.0814)	0.9999 (0.9946–1.0053)	0.9949 (0.9861–1.0038)	0.9971 (0.9862–1.0081)
*35–49 years*	1.0015 (1.0011–1.0019)*	0.8832 (0.7777–1.0030)	0.9984 (0.9709–1.0266)	1.0029 (1.0000–1.0059)	1.0010 (0.9963–1.0057)	0.9972 (0.9914–1.0030)
*50–64 years*	1.0022 (1.0017–1.0026)*	0.8059 (0.6946–0.9350)*	1.0136 (0.9815–1.0468)	1.0057 (1.0023–1.0091)*	1.0012 (0.9960–1.0064)	1.0020 (0.9956–1.0084)
*65+ years*	1.0010 (1.0005–1.0015)*	0.7407 (0.6207–0.8837)*	1.0332 (0.9949–1.0729)	1.0031 (0.9991–1.0071)	1.0080 (1.0016–1.0144)*	1.0082 (1.0004–1.0161)*

CI – confidence interval, IRR – incidence rate ratio

*Estimates are significant at *P* < 0.05.

Although with different magnitudes, the trends reveal that the weekly number of medication collection visits for all three chronic diseases fell significantly across all age groups for both males and females – except males aged 35 to 49 years – following the introduction of level 5 lockdown (**Table 2**). The weekly number of medication collection visits thereafter increased gradually for all three chronic diseases during the first COVID-19 wave (with lockdown levels 3–1). With a rate of increase of 0.62%, IRR = 1.0062 (95% CI = 1.0051–1.0074) per week, by the end of the first wave lockdown period, the weekly number of medication collection visits for HIV/AIDS reached the expected level based on the rate of increase experienced during the pre-COVID-19 period. However, despite also increasing, the weekly number of medication collection visits for hypertension and diabetes over the same period fell below the expected levels.

The trends further show that the weekly number of medication collection visits for HIV/AIDS and hypertension in 2021 (waves 2 and 3) were lower than those observed during the first wave lockdown level 3–1 period in 2020. Hence, during the third wave of the COVID-19 pandemic the weekly number of medication collection visits for HIV/AIDS and hypertension were far below the levels expected if the rate of weekly increase observed during the pre-COVID-19 period had continued. In contrast, for diabetes, after a drop during the second wave of the COVID-19 pandemic the weekly number of medication collection visits increased during the third wave to the level expected if the rate of weekly increase experienced during the pre-COVID-19 period had continued.

**Figure 3:**
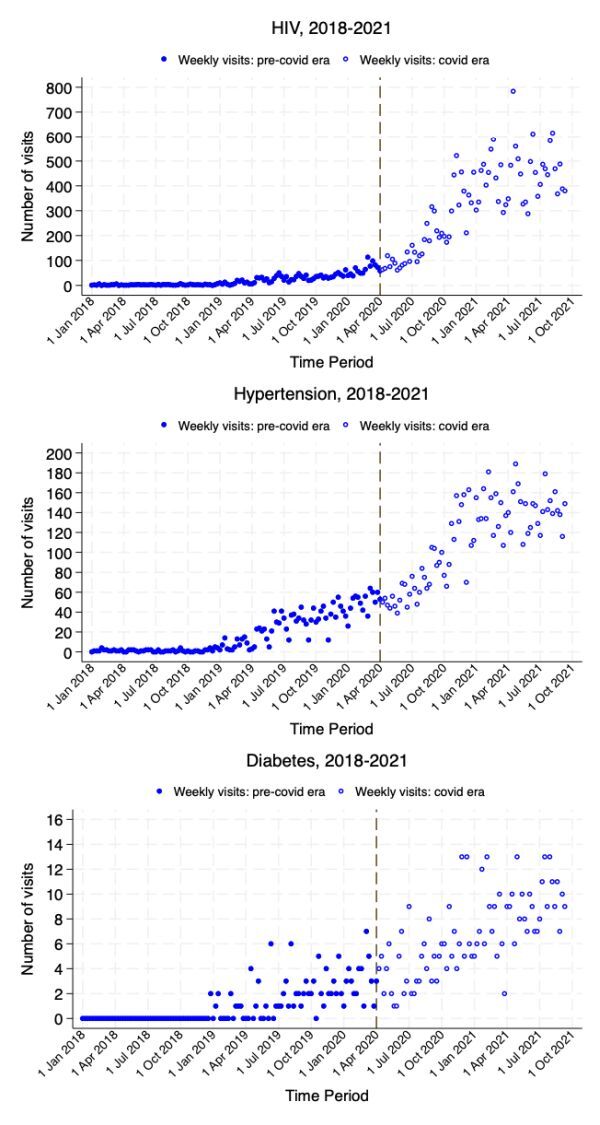
Trends in weekly number of Central Chronic Medicines Dispensing and Distribution (CCMDD) scheduled visits for medication collection for HIV/AIDS, hypertension and diabetes, Agincourt 2018–2021.

As shown in **Figure 3**, the decrease in the number of medication collection visits at clinics for HIV/AIDS, hypertension and diabetes observed in 2021 (**Figure 2**) can be in part attributed to the exponential increase in the number of medication collection visits from the CCMDD programme. For example, the average number of CCMDD scheduled visits for HIV/AIDS medication steadily increased from slightly over 100 per week in March 2020 before the emergence of the COVID-19 pandemic to about 500 per week by September 2021.

**Figure 4:**
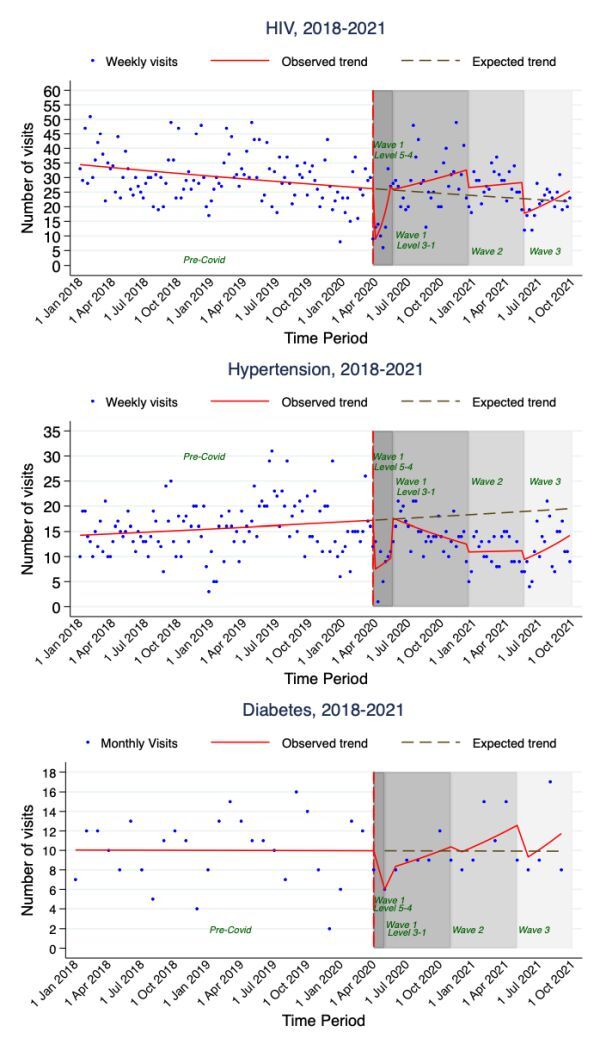
Trends in number of new chronic disease diagnostic visits by condition.

**Figure 4** and **Table 3** show trends in the number of new diagnoses for HIV/AIDS, hypertension and diabetes at periods from 1 January 2018 to 30 September 2021 in Agincourt primary health care facilities. During the pre-COVID-19 pandemic period, new diagnoses for HIV/AIDS were declining at a rate of 0.24% per week for HIV/AIDS, IRR = 0.9976 (95% CI = 0.9967–0.9986) and increasing at a rate of 0.16% per week for hypertension, IRR = 1.0016 (95% CI = 1.0003–1.0030). The number of new diagnoses for diabetes during the pre-COVID-19 pandemic period was stable. As expected, the number of new diagnoses for all three chronic diseases significantly dropped immediately after the introduction of level 5 lockdown in March 2020. The weekly number of new diagnoses for HIV fell immediately by 71.63%, IRR = 0.28 (95% CI = 0.17–0.46) and for hypertension by 57.94%, IRR = 0.42 (95% CI = 0.23-076). Changes in the number of new diagnoses for diabetes were assessed monthly given smaller numbers and even though there was a decrease at the onset of the COVID-19 pandemic, these changes were insignificant.

**Table 3:** Trends in number of new chronic disease diagnoses by condition, sex and age, Agincourt 2018–2021

Variables	Pre-lockdown trend, IRR (95% CI)	Immediate change at the start of lockdown, IRR (95% CI)	Level 5–4 trend, IRR (95% CI)	Level 3–1 trend, IRR (95% CI)	Second wave lockdown trend, IRR (95% CI)	Third wave lockdown trend, IRR (95% CI)
	exp*(β_1_)*	exp*(β_2_)*	exp*(β_1_* + *β_3_)*	exp*(β_1_* + *β_3_* + *β_5_)*	exp*(β_1_* + *β_3_* + *β_5_* + *β_7_)*	exp*(β_1_* + *β_3_* + *β_5_* + *β_7_* + *β_9_)*
HIV/AIDS	0.9976 (0.9967–0.9986)*	0.2837 (0.1742–0.4620)*	1.2019 (1.0931–1.3214)*	1.0079 (1.0002–1.0157)*	1.0030 (0.9905–1.0157)	1.0203 (1.0023–1.0386) *
Hypertension	1.0016 (1.0003–1.0030)*	0.4206 (0.2326–0.7606)*	1.0426 (0.9185–1.1835)	0.9884 (0.9778–0.9990)*	1.0011 (0.9814–1.0211)	1.0230 (0.9986–1.0480)
Diabetes	0.9997 (0.9853–1.0143)	0.5769 (0.2475–1.3446)	1.0438 (0.8952–1.2172)	1.0438 (0.8952–1.2172)	1.0499 (0.9123–1.2083)	1.0793 (0.8230–1.4155)
Females						
*18–34 years*	0.9987 (0.9972–1.0001)	0.2442 (0.1145–0.5209)*	1.1906 (1.0265–1.3811)*	1.0075 (0.9960–1.0190)	0.9997 (0.9812–1.0185)	1.0284 (1.0025–1.0549)*
*35–49 years*	1.0003 (0.9986–1.0020)	0.5024 (0.2541–0.9933)*	1.0856 (0.9426–1.2502)	0.9991 (0.9866–1.0119)	0.9950 (0.9722–1.0184)	1.0280 (0.9970–1.0601)
*50–64 years*	0.9995 (0.9975–1.0016)	0.4711 (0.1959–1.1326)	1.0552 (0.8762–1.2707)	0.9919 (0.9752–1.0088)	1.0082 (0.9791–1.0382)	1.0144 (0.9738–1.0567)
*65+years*	0.9998 (0.9970–1.0025)	0.4639 (0.1369–1.5724)	1.0364 (0.7973–1.3473)	0.9803 (0.9583–1.0029)	0.9799 (0.9394–1.0222)	1.0054 (0.9480–1.0664)
Males						
*18–34 years*	0.9978 (0.9948–1.0008)	0.2058 (0.0421–1.0069)	1.3024 (0.9693–1.7500)	1.0072 (0.9823–1.0327)	0.9976 (0.9558–1.0413)	1.0130 (0.9499–1.0803)
*35–49 years*	0.9987 (0.9964–1.0010)	0.4465 (0.1653–1.2062)	1.1108 (0.9069–1.3606)	1.0138 (0.9953–1.0327)	0.9932 (0.9626–1.0247)	1.0000 (0.9559–1.0461)
*50–64 years*	0.9971 (0.9943–0.9999)*	0.1432 (0.0235–0.8711)*	1.3024 (0.9300–1.8240)	0.9980 (0.9771–1.0193)	1.0136 (0.9814–1.0469)	1.0274 (0.9795–1.0776)
*65+ years*	1.0000 (0.9962–1.0039)	0.1700 (0.0144–2.0039)	1.1350 (0.6886–1.8710)	1.0060 (0.9754–1.0375)	1.0084 (0.9585–1.0610)	1.0230 (0.9520–1.0993)

CI – confidence interval, IRR – incidence rate ratio

*Estimates are significant at *P* < 0.05.

During subsequent periods of the pandemic, the number of new diagnoses for HIV/AIDS increased significantly, surpassing the level expected if the rate of weekly change observed during the pre-COVID-19 period had continued. In contrast, despite some recovery in the number of new diagnoses of hypertension following an initial major fall during levels 5–4 of the first wave lockdown, they did not reach the level expected if the rate of weekly increase experienced during the pre-COVID-19 period had continued. For diabetes, the number of new diagnoses during the first wave lockdown levels 3–1 and second and third wave periods fell within the pre-COVID trend bounds expected. However, given small numbers of new diagnoses, the trends are difficult to interpret.

There were notable differences in the number of new diagnoses and trends for the three major chronic diseases among females and males. However, the trends should be interpreted with caution due to the small numbers especially in the older age groups.

## DISCUSSION

This study provides unique insights into shifts in health care access and service provision for three major chronic diseases – HIV/AIDS, hypertension and diabetes – in a rural South African community during the first three waves of the COVID-19 pandemic. It highlights notable declines in visits for medication collection for all three chronic diseases immediately after the introduction of level 5 lockdown during the first wave of the pandemic. It also shows that although clinic visits increased during later stages of the pandemic, by the end of the third wave in September 2021, the weekly number of visits for HIV/AIDS and hypertension medication collection remained below the levels that would have been expected had the pre-COVID-19 trends continued uninterrupted. Furthermore, it shows considerable declines in new diagnoses for hypertension, and HIV/AIDS at the beginning of the pandemic, with only HIV/AIDS diagnoses catching up to pre-pandemic levels by the end of the third wave. Additionally, despite the reduction in the number of clinic visits, the study findings suggest that most patients with pre-existing chronic conditions likely continued to access care and collect their medications as many were progressively transitioned to the CCMDD programme, which allowed them to collect medications without visiting clinics.

Traditionally, with a focus on HIV/AIDS medication, enrolment into the CCMDD programme required meeting strict prerequisites, where only patients with well-controlled chronic conditions were eligible. Therefore, the exponential increase in numbers of chronic disease patients enrolled into the programme over the COVID-19 pandemic period raises concerns about whether adherence to the eligibility criteria remained stringent, particularly in relation to comorbidity control. Given the uncertainty surrounding COVID-19 infection risk, the anticipated strain on health care services, and unknowns related to co-infection and interaction with HIV, it is plausible that less rigorous criteria may have been applied to some patients enrolled in the CCMDD programme during the pandemic period. The scale of this concern remains unknown, and further studies are required to assess it along with its impact on long-term health outcomes.

Concerns around the quality of patient care during the pandemic have been raised by findings from a review of National Health Laboratory Services data, which showed that the weekly number of tests had reduced by 22% for HIV-1 viral load and 33% for CD4+ T cell during the first level 5 lockdown, highlighting significant compromises in the quality of care received for HIV [13]. It is also critical to investigate the extent to which the reduction in the absolute number of medication collection visits was due to patients lost to follow-up. This is particularly important given findings from other studies highlight numerous barriers in accessing care during the COVID-19 pandemic, such as fear of contracting COVID-19 at health care facilities, financial constraints, longer waiting times, and altered clinic operations due to new staffing protocols and screening measures [22,23].

The reduction in new HIV/AIDS diagnoses following the implementation of the level 5 lockdown during the first wave of the pandemic was consistent with findings from a study in KwaZulu-Natal, which reported a 50% reduction in HIV testing and ART initiation during that period [12]. These reductions likely reflect a sharp decline in routine HIV screening and testing services. However, after this significant drop, our study observed a sharp increase in new HIV/AIDS diagnoses, surpassing pre-COVID-19 pandemic levels. Similarly, the study in KwaZulu Natal also showed a gradual recovery in new HIV/AIDS diagnoses, returning to pre-COVID-19 pandemic levels by July 2020 [12].

The initial level 5 lockdown was implemented pre-emptively, allowing health care facilities to prepare, at a time when national COVID-19 cases were low and no local cases had been detected in our study area in Mpumalanga Province. Our findings suggest that after this preparatory phase and accompanying initial decline in visits for new diagnoses, HIV services responded robustly. However, hypertension services did not receive the same level of priority, likely due to the long-standing focus on HIV services over noncommunicable diseases in primary health care settings. Given rising morbidity and mortality associated with hypertension in South Africa, there is an urgent need to enhance hypertension screening in primary health care facilities.

Finally, our study findings further highlight the well-established disparity in clinic attendance by sex, with men consistently attending fewer visits for all the disease conditions assessed, despite these diseases not being sex-specific. For example, in the Agincourt HDSS, among individuals aged 40 years and older, the prevalence of HIV is 23% for males and 22.9% for females, hypertension is 58.4% for males and 67.1% for females, and diabetes is 10.5% for males and 12.4% for females. Nevertheless, our study findings do not indicate any significant differential impact of the COVID-19 restrictions on clinic attendance by sex or gender.

### Limitations

The decrease in chronic disease screenings could have been as a result of the reduced provision of health care services for non-COVID-19 conditions. However, scheduled follow-up medication collection visits were unlikely affected by such reductions. Additionally, as data used in our study were collected exclusively within health care facilities, we did not assess whether patients scheduled to collect their medication from the CCMDD programme did subsequently do so. With fear driving poor clinic attendance, this may have resulted in lower-than-expected medication collection visits. Finally, the small sample size for diabetes-related visits limit the conclusions we can draw, but we hypothesise that the trends for diabetes visits would align with those observed for hypertension.

## CONCLUSIONS

The COVID-19 pandemic placed immense strain on already overburdened health care systems worldwide, underscoring the importance of accurate data on health care utilisation and provisional to inform effective responses. Our findings indicate that, despite the initial disruptions to HIV-related care at the clinic level, services were able to recover and meet patient demand. The study also highlights the potential of programmes like CCMDD in maintaining access to care for chronic diseases other than HIV. However, it also reveals that hypertension screening and diagnosis was significantly affected by the pandemic and was yet to return to pre-pandemic levels by the end of the third wave of the COVID-19 pandemic in South Africa. It is essential for local health authorities to assess the continued effects of COVID-19 and other pandemic control measures on access to health care for non-COVID-19 related conditions. By doing so, they can develop tailored strategies to mitigate the severe unintended opportunity costs these disruptions had on patients’ health.
